# Unrevealing the therapeutic potential of artesunate against emerging zoonotic *Babesia microti* infection in the murine model

**DOI:** 10.3389/fvets.2024.1383291

**Published:** 2024-05-09

**Authors:** Saqib Ali Fazilani, Wei An, Sihong Li, Mohammad Farooque Hassan, Muhammad Ishfaq, Shakeel Ahmed Lakho, Muhammad Farooque, Muhammad Shoaib, Xiuying Zhang

**Affiliations:** ^1^Heilongjiang Key Laboratory for Animal Disease Control and Pharmaceutical Development. Faculty of Basic Veterinary Science, College of Veterinary Medicine, Northeast Agricultural University, Harbin, China; ^2^Department of Veterinary Pharmacology and Toxicology, Faculty of Biosciences, Shaheed Benazir Bhutto University of Veterinary and Animal Sciences, Sakrand, Pakistan; ^3^Technical Centre of Chengdu Customs, Chengdu, China; ^4^College of Animal Science and Technology and College of Veterinary Medicine of Zhejiang A&F University, Hangzhou, China; ^5^Department of Veterinary Pathology, Faculty of Veterinary Sciences, Shaheed Benazir Bhutto University of Veterinary and Animal Sciences, Sakrand, Pakistan; ^6^Huanggang Normal University, Huanggang, China; ^7^Department of Veterinary Parasitology, Faculty of Veterinary Sciences, Shaheed Benazir Bhutto University of Veterinary and Animal Sciences, Sakrand, Pakistan; ^8^Faculty of Veterinary and Animal Sciences, Ziauddin University Karachi, Karachi, Pakistan; ^9^Key Laboratory of New Animal Drug Project, Gansu Province/Key Laboratory of Veterinary Pharmaceutical Development, Ministry of Agriculture and Rural Affairs, Lanzhou Institute of Husbandry and Pharmaceutical Sciences of Chinese Academy of Agriculture Sciences, Lanzhou, China

**Keywords:** artesunate, babesiosis, histopathology, blood-borne pathogen, apoptosis, immune response, inflammation

## Abstract

Babesiosis, a zoonotic blood protozoal disease, threatens humans and animals and is difficult to treat due to growing antimicrobial resistance. The study aimed to investigate the therapeutic efficacy of artesunate (AS), a well-known derivative of artemisinin, against *Babesia microti* (*B. microti*) using a murine infection model. Male BALB/c mice (6 weeks old; 15 per group) were chosen and randomly divided into 1) the control group, 2) *the B. microti* group, and 3) the *B. microti* + artesunate treatment groups. AS treatment at 2 mg/kg, 4 mg/kg, and 8 mg/kg of body weight significantly (*p* < 0.05) reduced the *B. microti* load in blood smears in a dose-dependent manner. Additionally, AS treatment mitigated the decrease in body weight and restored the normal state of the liver and spleen viscera index compared to the *B. microti*-infected group after 28 days. Hematological analysis revealed significant increases in RBC, WBC, and PLT counts post-AS treatment compared to the *B. microti*-infected group. Furthermore, AS administration resulted in significant reductions in total protein, bilirubin, ALT, AST, and ALP levels, along with reduced liver and spleen inflammation and lesions as observed through histopathological analysis. AS also elicited dose-dependent changes in mRNA and protein expression levels of apoptotic, proinflammatory, and anti-inflammatory cytokines in the liver compared to the control and *B. microti*-infected groups. Immunolabeling revealed decreased expression of apoptotic and inflammation-related proteins in AS-treated hepatic cytoplasm compared to the *B. microti*-infected group. AS also in dose-dependent manner decreased apoptotic protein and increased Bcl-2. Overall, these findings underscore the potential of AS as an anti-parasitic candidate in combating *B. microti* pathogenesis in an *in vivo* infection model, suggesting its promise for clinical trials as a treatment for babesiosis.

## 1 Introduction

Babesiosis, a protozoal infection, manifests as a parasitic condition capable of infecting diverse vertebrate hosts, including penguins, pigs, and humans. Recently, there has been a notable rise in zoonotic *Babesia* infections, and the number of species capable of infecting humans consistently rising (1). It is becoming more common, and this raises concerns in the veterinary and medical fields. *B. microti* primarily disseminates through rodents and small mammals in North America and Asia. Over the past two decades, over a hundred cases of *B. microti* infection have been identified in different provinces across China, which establishes that this protozoal pathogen is the main source of babesiosis in the Chinese population (2).

The replication of the parasite within the red blood cells (RBCs) leads to the manifestation of clinical indicators, such as fever, hemolytic anemia, loss of appetite, hemoglobinuria, and considerable weight loss. In more severe instances of infection, the affected individual may culminate in a fatality, thus causing significant global losses (3, 4). The incubation period starts after a tick bite primarily, and it ranges from 1 to 6 weeks (5). However, in conditions when this *B. microti* has been transmitted through a blood transfusion, the highest severity of parasitemia can appear within a short period and leads to a wide range of clinical manifestations, including acute respiratory distress syndrome, disseminated intravascular coagulation, congestive heart failure, and renal failure (6). The currently available therapeutic options for *B. microti* are limited, and the development of drug resistance warrants immediate consideration (4). Considering the growing increase in antimicrobial resistance, it is difficult to treat, and there is an urgent need for alternative treatments that can specifically target this diverse parasite (7).

Artesunate (AS) is alternatively referred to as dihydroartemisinin-12-α-succinate and belongs to the category of semisynthetic compounds characterized by a peroxide-bridged sesquiterpene lactone structure (8). This compound is the biologically active constituent in the traditional Chinese medicinal plant, *Artemisia annua*. Earlier, AS was an antimalarial drug and primarily used to treat malaria, but its effectiveness against other protozoan parasites, like *Babesia,* was also observed (9, 10). AS gained much attention from researchers due to its unique pharmacological properties against several diseases. In addition to its antimalarial activity, this drug showed efficacy against several diseases caused by viruses, bacteria, and protozoa. Studies showed the potential effect of AS on SARS-Covid-1, Covid-2, and Covid-19 (11), schistosomiasis (12), leishmaniasis (13), babesiosis (9), tuberculosis, and many livestock diseases (14). Studies showed that AS also has immunomodulatory effects on tumor necrosis factor-alpha (TNF-α) and interleukin-6 (IL-6) in rats (15). A previously conducted study showed that AS treatment in sepsis-induced lung injury showed attenuated caspase levels and decreased apoptotic protein expression (16).

To date, the effects of AS on experimental babesiosis in mice infection models have not been studied in detail at the histopathologic and apoptotic molecular levels. This study provides substantial evidence for this purpose and uncovers the potential effects of the drug on the pathogenicity of *Babesia* parasites. In this study, the therapeutic potential of AS in inhibiting the pathogenesis of babesiosis was evaluated by hematology, microscopy, and biochemistry analysis along with histopathological observation, immunohistochemistry analysis, real-time quantitative PCR (qPCR), and Western blot analysis to explore the molecular mechanisms of disease progression and treatment response. Additionally, the molecular and physiological aspects of *Babesia* pathogenesis and the potential ameliorative effect of artesunate therapy were studied.

## 2 Materials and methods

### 2.1 Experimental animals and parasites

The mice testing was approved by the Animal Care and Use Committee of Northeast Agricultural University; 6-week-old male mice BALB/c (weight: 0.032 ± 0.001 g/kg) were selected and purchased from Liaoning Changsheng Biotech (Benxi, Liaoning, China) for this study, and a confirmed *B. microti* strain was obtained via performing a PCR test (reference strain, ATCC 30221). Artesunate was purchased from MedChem Express, China, for intramuscular administration in mice. Before the treatment, all mice were acclimatized and housed in a 12-h/12-h light/dark cycle at 23°C with fresh water and *ad-libitum* feeding. After a 1-week adaptation period, the experimental mice were randomly divided into five groups (15 per group) as follows: (1) the control group (CG), (2) the *B. microti* group (BM), (3) the *B. microti* + artesunate (2 mg/kg) low-dose group (LG), (4) the *B. microti* + artesunate (4 mg/kg) medium-dose group (MG), and (5) the *B. microti* + artesunate (8 mg/kg) high-dose group (HG). The different concentrations of drugs were prepared by following the method of Carvalho et al. (17). The *B. microti* was intraperitoneally administered to mice with 1 × 10^7^ parasites following the method (9). After induction of *B. microti* infection reached 30 to 40%, mice were treated with AS daily for 4 weeks, and blood smear microscopy was used to analyze the parasites every second day. Blood samples were collected for routine blood biochemistry analysis on weekly basis. The body weight was measured every 3 day and final measured at 28th day of study. After 4 weeks, all groups were euthanized, and organs (liver and spleen) were collected and processed for further experiments following the standard protocols.

### 2.2 Blood smear examination

The detection of *B. microti* in blood samples was carried out by following the methodology outlined in prior research (18). According to the Centers for Disease Control (CDC) and the World Health Organization (WHO), the blood smear is still the preferred and fastest method for the detection of *B. microti* parasite in blood (19). Blood smear films were allowed to air dry. Subsequently, they were fixed through immersion in absolute methanol for 1 to 2 s. Following fixation, the films were left to dry at room temperature. A staining protocol was adopted, involving a 30% Giemsa solution (Abcam, China) application to the blood films for 15 min, maintaining a temperature of approximately 20°C. Upon completing the staining process, the slides were meticulously washed and dried before examination under a compound microscope (Nikon Instrument, Inc., Japan). This microscopic analysis employed a “100 X” lens and an immersion oil drop (20).

### 2.3 Hematology and blood profiling

The blood profile of red blood cells (RBCs), white blood cells (WBCs), and platelets (PLTs) were determined through Mindray BC-5000 Vet Auto Hematology Analyzer, Shenzhen Mindray Animal Medical Technology Co., Ltd., China, following the guidelines of the manufacturer.

### 2.4 Blood biochemical parameters of the liver

The determination of liver biochemical parameters includes total protein concentration, bilirubin concentration, as well as the activities of critical enzymes such as serum alanine transaminase (ALT), aspartate transaminase (AST), and alkaline phosphatase (ALP). Blood samples were collected from the tail vein of mice using a 25-gauge needle and centrifuged at 3,000 rpm for 10 min to separate serum. The serum was transferred to a clean tube for enzymatic analysis according to the manufacturer’s kits method (Jiangsu Aidisheng Biological Technology Co., Ltd).

### 2.5 Histopathological observation

Hematoxylin–eosin (HE) staining was performed using the previous method (21). Specifically, the liver and spleen tissues were fixed with 4% paraformaldehyde, dried with gradient alcohol, cleared with dimethylbenzene, and then embedded in paraffin. A rotary microtome (Leica, Germany) was then used to slice the tissues into 5 μm thin slices. The tissue sections were inspected under a microscope using a Leica Aperio CS2 slide scanner (Wetzlar, Germany).

### 2.6 Quantitative real-time PCR (qRT-PCR)

Liver samples were used to extract total RNA, and the concentration and quantity of the extracted RNA were measured using a NanoDrop 2000 spectrophotometer (Thermo Fisher Scientific, United States) at 260 nm and 280 nm, respectively. RNA with absorbances between 1.8 and 2.1 at A260/A280 were utilized for the study. Then, first-strand cDNA was synthesized utilizing a method previously established by our research team (21). With the help of Primer 5.0 software, specific gene primers were made for targets like interleukin-2 (IL2), interferon-gamma (IFN-γ), Beclin 1, tumor necrosis factor-alpha (TNF-α), CD44, caspase-9, and Bax. As a reference for normalization, GAPDH was employed as a housekeeping gene. Specific gene primers and housekeeping gene primers for qPCR is given in Table 1. The qPCR process was executed utilizing the FastStart Universal SYBR Green Master (ROX) Kit from Roche, Shanghai, China. A pre-denaturation step at 95°C for 10 min was the first step in the amplification process. This was followed by 40 cycles of denaturation at 95°C for 15 s, annealing at 58°C for 30 s, extension at 95°C for 15 s, and final extension at 37°C for 30 s. The 2^-∆∆CT^ method was used for mRNA expression levels (22, 23).

**Table 1:** Genes and their primer information used for qRT-PCR analysis.

Sr. No.	Genes	Primers (5′-3′)
1	IL-2	Forward: GGATCCATGATGTGCAAAGTACTG Reverse: CGGTCGACTTATTTTTGCAGATATCT
2	IFN- γ	Forward: GGATCCATGATGTGCAAAGTACTG Reverse: CGGTCGACTTATTTTTGCAGATATCT
3	Beclin 1	Forward: CGACTGGAGCAGGAAGAAG Reverse: TCTGAGCATAACGCATCT
4	TNF- α	Forward: CCG AGG CAG TCA GAT CAT CTT Reverse: AGC TGC CCC TCA GCT TGA
5	CD44	Forward: CCAGAAGGAACAGTGGTTTGGC Reverse: ACTGTCCTCTGGGCTTGGTGTT
6	Caspase 9	Forward: GTTTGAGGACCTTCGACCAGCT Reverse: CAACGTACCAGGAGCCACTCTT
7	Bax	Forward: TCAGGATGCGTCCACCAAGAAG Reverse: TGTGTCCACGGCGGCAATCATC
8	GAPDH	Forward: GCACGCCATCACTATCTT Reverse: GGACTCCACAACATACTCAG

### 2.7 Immunohistochemistry analysis

For immunohistochemistry staining, the liver tissues were first fixed in a solution with 4% paraformaldehyde; they were then embedded in paraffin. Sections of 4 μm thickness were then cut from these paraffin-embedded tissues. The antigen repair solution with citric acid (pH 6.0, Service Bio, China) was employed to enhance antigen retrieval. After this step, the slides were treated with a 10% rabbit serum (Service Bio) to block non-specific binding. The slides were placed in primary antibody (Table 2) and stored at 4°C overnight. Following this, the slices were subjected to DAB (3,3′-diaminobenzidine) treatment. Subsequently, the slides were dehydrated using alcohol and subjected to hematoxylin (Service Bio, China) staining. The stained slides were examined under a microscope (Nikon E-100 Tokyo, Japan). Images were captured using the Case Viewer program (3DHISTECH, Hungary Ltd.).

**Table 2:** Primary antibodies, their manufacturers, and the dilution ratio used for Immunohistochmeistry analysis.

Primary antibody	First anti-item number	Primary antibody manufacturer	The dilution ratio of one antibody
TNF-α	bs-10802R	Bioss	1:400
Caspase 9	A2636	ABclonal	1:200
Bax	AY0553	Always	1:200
CD44	ab189524	Abcam	1:200

### 2.8 Western blotting analysis

Total proteins were extracted following our previous study (24). Then, protein samples were subjected to a series of steps following a previous technique in our laboratory (25). These include protein processing, electrophoresis, membrane transfer, containment, and antibody incubation. The protein bands were determined through a Hypersensitive ECL luminous solution kit (MA0186, Meilun Bio, Dalian, China), and the images were acquired by Tanon (Tanon Life Sciences Ltd. Shanghai, China). The optical density of the protein band was measured by using ImageJ software from the National Institutes of Health, United States. To ensure consistency, the relative protein expression levels were normalized to GAPDH, a housekeeping protein. The antibodies employed in this process are listed in Table 3.

**Table 3:** Antibodies used in Western Blotting analysis, their product, and sourcing details.

No.	Antibodies	Product & source information
1	Bcl-2	Bcl-2; 1:500; Catalogue# HY-p80030, Med Chem Express, China
2	BAX	BAX; 1:500; Catalogue# HY-122760, Med Chem Express, China
3	ICAM-1	ICAM-1; 1:2000; Catalogue# HY-P80502; Med Chem Express, China
4	SICAM-1	Anti-ICAM-1; 1:250; Catalogue# AB171123; Abcam, China
5	CRP	Anti-CRP; 1:1000; Catalogue# FNab01995; FN-test, China
6	GAPDH	Anti-GAPDH; 1:5000; Catalogue# HY-P80137; Med Chem Express, China

### 2.9 Statistical analysis

To ensure accuracy and reproducibility, all the above tests were repeated triplicate for validation. Statistical analysis was performed using GraphPad Prism (Ver. 9.0) with one-way variance. Data are presented as mean ± SEM, with *p*-values *<*0.05 were considered statistically significant using the Tukey’s method for post-hoc testing. Images were created with GraphPad Prism 9 (San Diego, CA).

## 3 Results

### 3.1 Effect of AS treatment on blood smear analysis of the *B. microti*-infected and treatment groups

**Figure 1:**
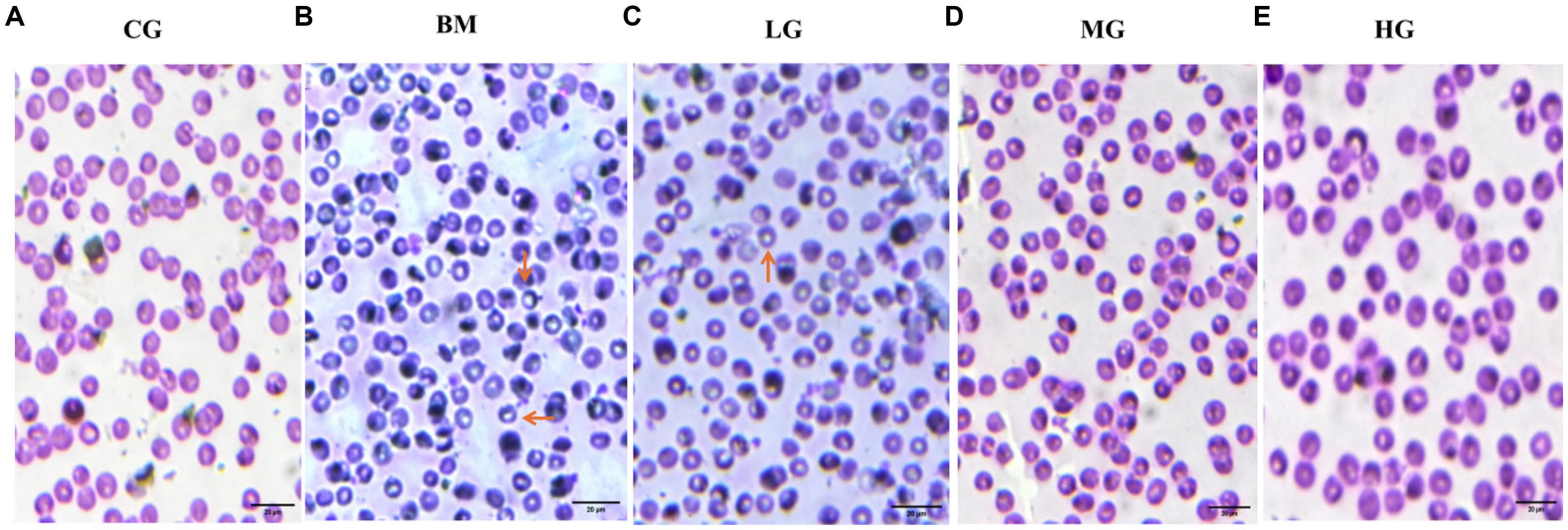
Results of Giemsa-stained thin blood smears (observed at 100X magnification). **(A)** The control group (CG), **(B)** the *B. microti* group (BM), **(C)** the *B. microti* + artesunate @2 mg/kg, low-dose group (LG), **(D)** the *B. microti* + artesunate @4mg/kg, medium-dose group (MG), **(E)** the *B. microti* + artesunate @8 mg/kg, high-dose group (HG). Arrows indicate the presence of *B. microti*, and scale bars represent 20 μm.

Blood smears were prepared and examined for each of the experimental groups, which included the control group (CG), the *B. microti*-infected group (BM), and the *B. microti*-infected groups treated with varying dosages of artesunate 2 mg/kg (LG), 4 mg/kg (MG), and 8 mg/kg (HG). Notably, blood smear results showed Figure 1 that AS in a dose-dependent manner reduced the *B. microti* load in the treatment groups, LG, MG, and HG, as compared to the BM group. This observation underscores the potential influence of AS at the specified dosage on the staining characteristics of blood smears, thereby warranting further investigation and analysis within the context of this study.

### 3.2 Influence of *B. microti* infection and AS treatment on body weight, liver, and spleen viscera

**Figure 2:**
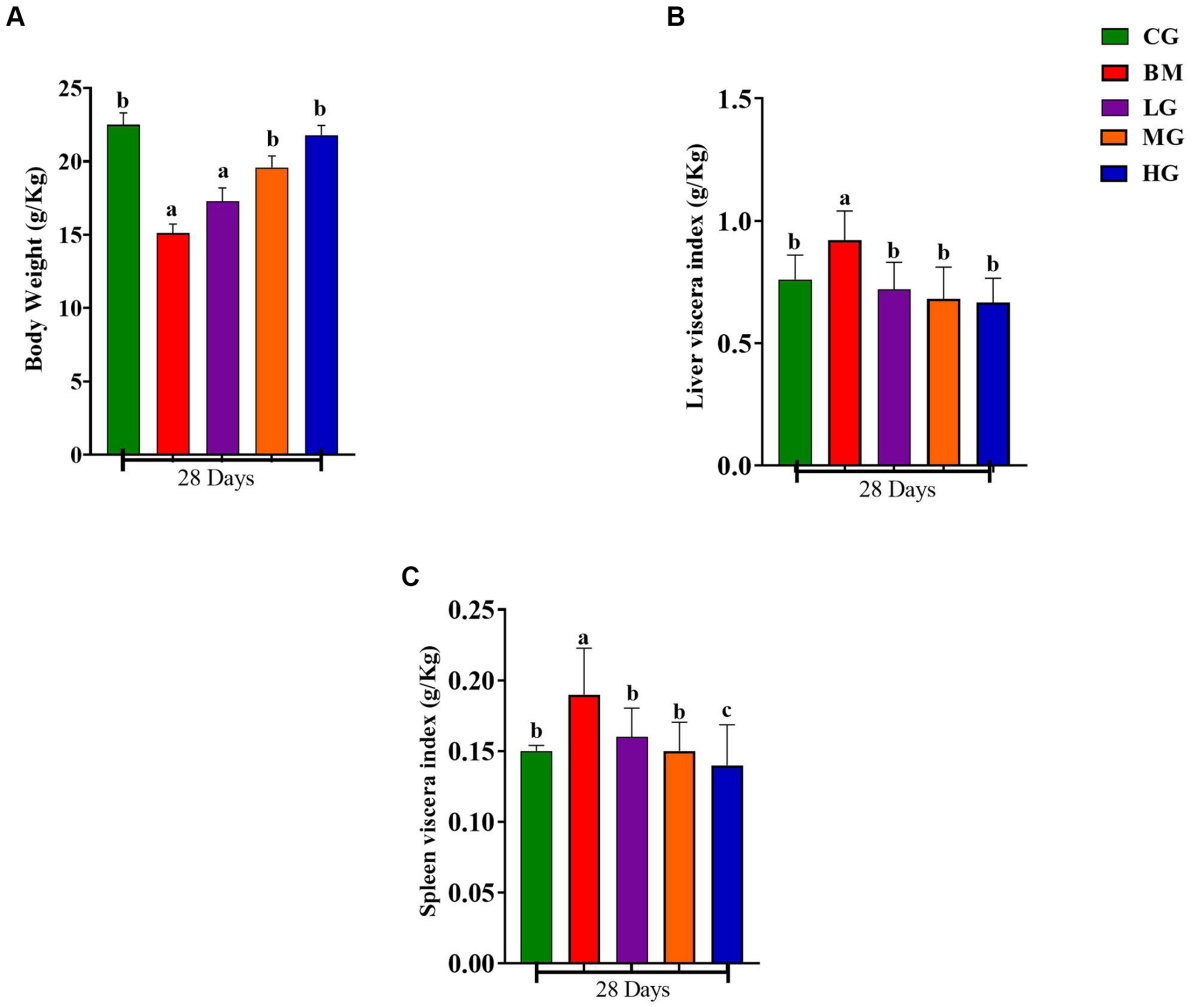
Results of body weight, liver, and spleen index. Experimental groups represented as the control group (CG), the *B. microti* group (BM), the *B. microti* + artesunate 2 mg/kg low-dose group (LG), the *B. microti* + artesunate 4 mg/kg medium-dose group (MG), and the *B. microti* + artesunate 8 mg/kg high-dose group (HG). **(A)** Results of body weight index. **(B)** Results of liver viscera index. **(C)** Results of spleen viscera index. Different lowercase letters represent significant differences between groups (*p* < 0.05).

In Figure 2, notable alterations in body weight, as well as in the liver and spleen viscera, were observed compared to the BM-infected group in mice on the 28th day of the study. Specifically, Figure 2A illustrates that a significant reduction of body weight in mice infected with BM compared with mice in the control group (CG) (*p* < 0.05) was evident. However, it is noteworthy that with the AS treatment 2 mg/kg (LG), 4 mg/kg (MG), and 8 mg/kg (HG) for 28 days, the body weight loss could be reversed and exhibited a recovery trend (*p < 0.05*).

Similarly, as depicted in Figures 2B,C, the weight of the liver and spleen viscera demonstrated a significant increase in mice infected with *B. microti* (BM) when compared to the control group (CG) (*p < 0.05*), whereas the liver and spleen viscera dimensions remained unchanged. Furthermore, a minor weight recovery was observed in mice treated with a dosage of 2 mg/kg (LG), while a substantial recovery was noted in mice treated with dosages of 4 mg/kg (MG) and 8 mg/kg (HG). These data underscore the potential therapeutic impact of AS on mitigating the observed reductions in body weight and organ viscera sizes induced by *B. microti* infection.

### 3.3 Effect of AS treatment on hematological parameters (WBC, RBC, and platelets)

**Figure 3:**
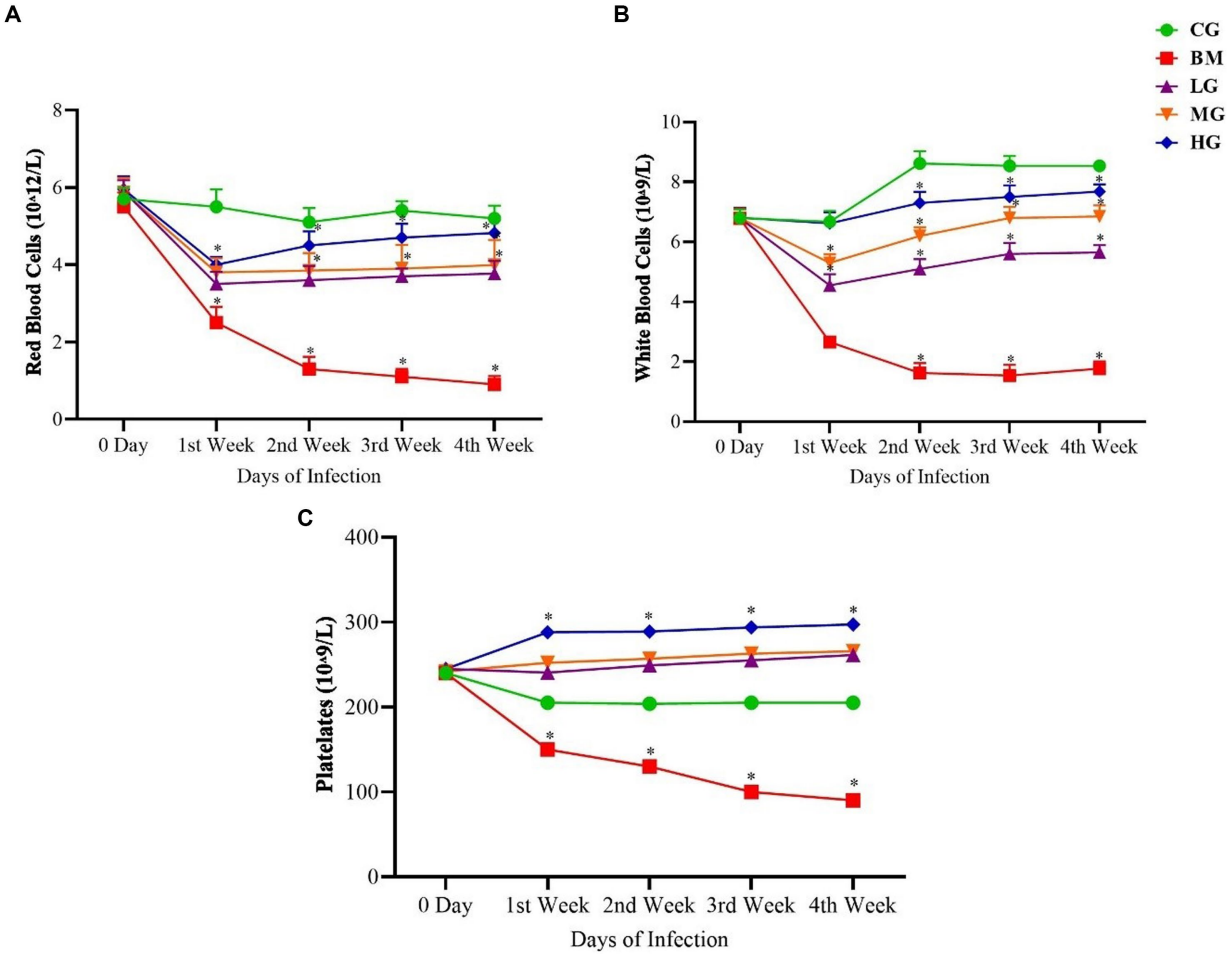
Results of **(A)** red blood cells (RBCs), **(B)** white blood cells (WBCs), and **(C)** platelets (PLTs). Experimental groups represented as the control group (CG), the *B. microti* group (BM), the *B. microti* + artesunate 2 mg/kg low-dose group (LG), the *B. microti* + artesunate 4 mg/kg medium-dose group (MG), and the *B. microti* + artesunate 8 mg/kg high-dose group (HG). The results are presented as means ± standard error of the mean (SEM). The data were analyzed, considering the significant differences with * (*p* < 0.05).

A comprehensive analysis of hematologic parameters was conducted between the groups, encompassing the CG, BM, and AS-treated LG, MG, and HG. The findings showed that comparative evaluation of red blood cell count (RBCs, Figure 3A), white blood cell count (WBCs, Figure 3B), and platelet count (PLTs, Figure 3C) exhibited distinct patterns. Within the *B. microti*-infected group (BM), a significant reduction was observed in the counts of RBCs, WBCs, and PLTs when contrasted with the control group (CG) (*p* < 0.05). This reduction signifies the hematologic impact of *B. microti* infection on these critical blood cell populations.

Conversely, a dose-dependent trend was observed following AS administration. Specifically, the groups LG, MG, and HG exhibited a notable and significant increase (*p < 0.05*) in the counts of RBCs, WBCs, and PLTs. This increment in blood cell counts underscores the potential of AS treatment to positively influence altered hematologic parameters of *B. microti* infection.

### 3.4 Influence of AS on liver biochemistry

**Figure 4:**
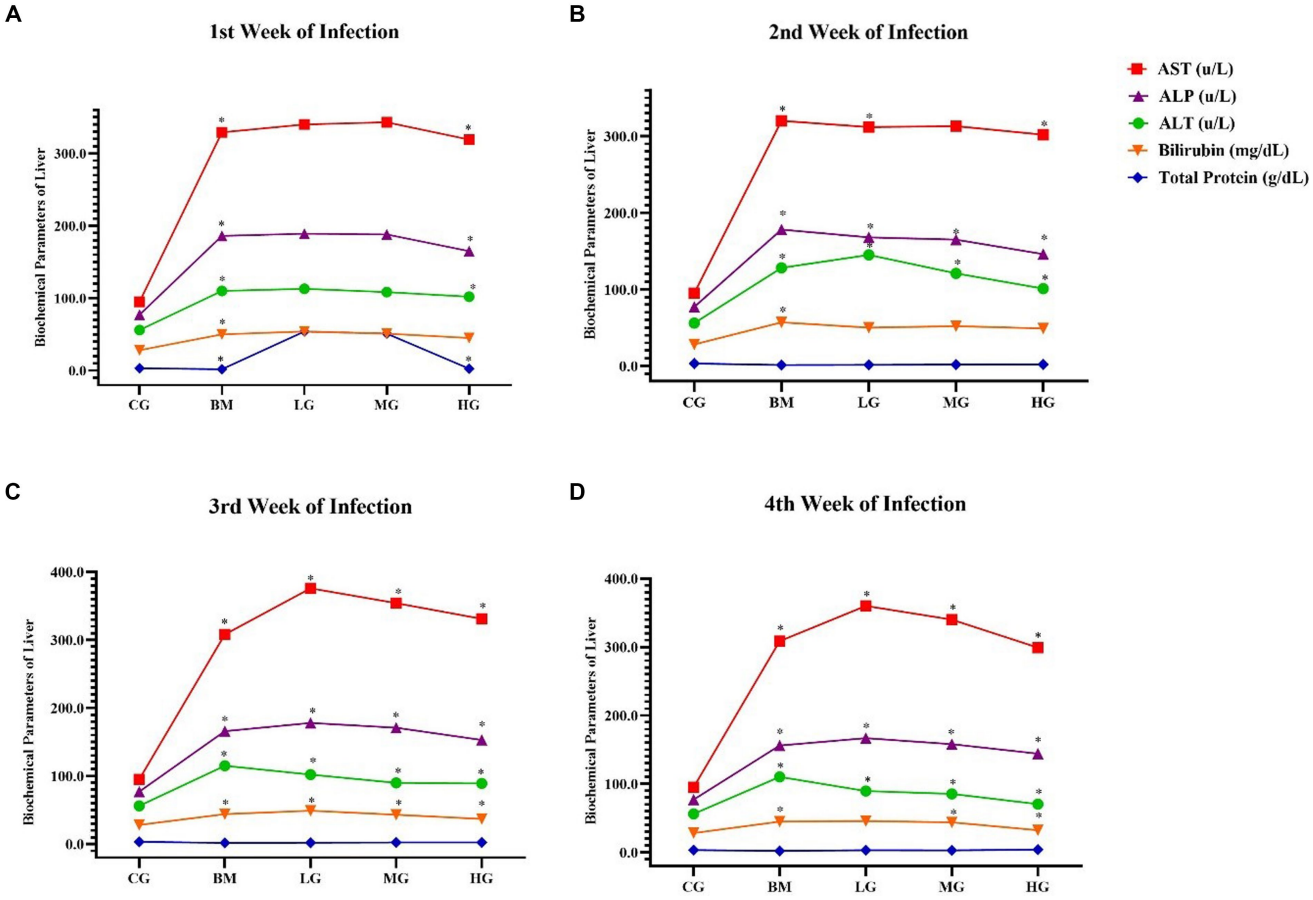
The results of blood biochemical parameters of the liver. Experimental groups, the control group (CG), the *B. microti* group (BM), the *B. microti* + artesunate 2 mg/kg low-dose group (LG), the *B. microti* + artesunate 4 mg/kg medium-dose group (MG), the *B. microti* + artesunate 8 mg/kg high-dose group (HG). **(A)** 1st week infection; **(B)** 2nd week infection; **(C)** 3rd week infection; and **(D)** 4th week of infection. The results are presented as means ± standard error of the mean (SEM). The collected data were analyzed, considering the significant differences with * (*p < 0.05*).

As shown in Figure 4, In contrast, the mice in the *B. microti*-infected (BM) group exhibited a significant (*p* < 0.05) increase in the concentration of aspartate transaminase (AST), Additionally, minor increases were observed in the levels of ALT, ALP, bilirubin, and total protein, compared to the control group (CG), throughout the 1st, 2nd, 3rd, and 4th weeks (*p* < 0.05). An increase in these blood biochemical parameters indicated liver injury.

Furthermore, it is noteworthy that mice treated with artesunate at dosages of 2 mg/kg (LG), 4 mg/kg (MG), and 8 mg/kg (HG) displayed minor decreases and recovered the elevated levels within the normal range as compared to the *B microti*-infected (BM) group (*p* < 0.05). These findings suggest that AS may play a role in mitigating the alterations in these biochemical parameters induced by *B. microti* infection.

### 3.5 Protective effect of AS treatment on histopathological changes in liver and spleen tissue

**Figure 5:**
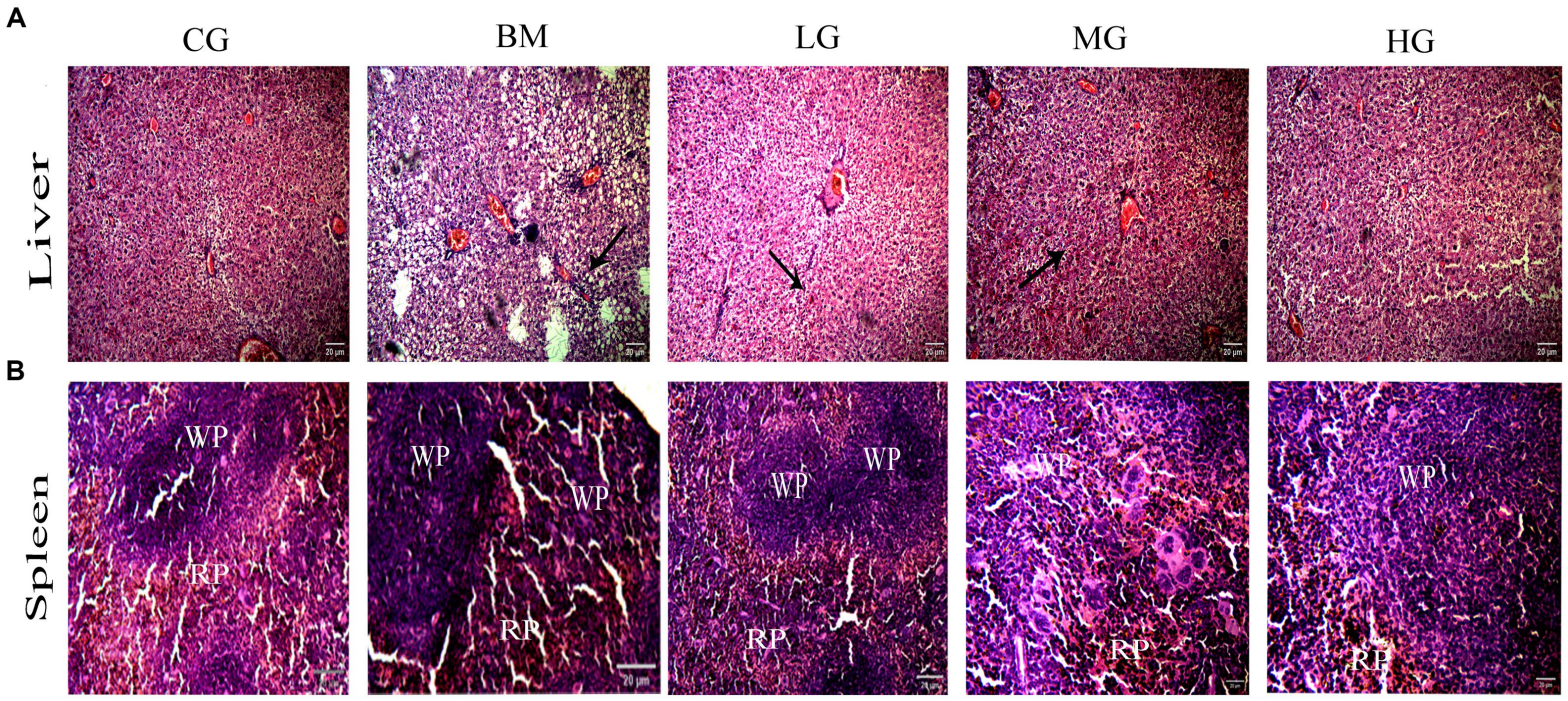
**(A)** Protective effect of AS treatment in liver on photomicrograph at 100 X magnification and scale bars 20 μm. Arrows show severe inflammatory lesions in the liver. **(B)** Effect of A S treatment on spleen photomicrograph at 100 X, Scale bar 20 μm. White pulp (WP) and red pulp (RP) structures in the *B. microti* group (BM). Recovery trend in the *B. microti* + artesunate 2 mg/kg low-dose group (LG), the *B. microti* + artesunate 4 mg/kg medium-dose group (MG), and the *B. microti* + artesunate 8 mg/kg high-dose group (HG) groups.

Our results showed that the liver histopathology of mice infected with *B. microti* (BM) (Figure 5A) exhibited moderate-to-severe inflammatory infiltrate and inflammatory lesions compared with the treatment group, this liver cellular inflammatory damage in the AS-treated groups significantly improved or recovered in a dose-dependent manner. The results here clearly indicate that hepatic lobules and central vein are unformed in appearance seen uniformly and well organized in the CG and HG groups to show that AS with a high dose of 8 mg/kg (HG) alleviated the liver damage and possess potent effect against *B. microti* infection.

Further, histopathology results of the spleen displayed a clear distinction between the red and white pulp structures, and marginal zones in the control group (CG) (Figure 5B). While the *B. microti* (BM) infection group showed serious congestion and enlarged red pulp. In a dose-dependent manner, we observed a recovered state of spleen cellular structure with weak red pulp cellularity and white pulp with clear marginal zones around follicles in the infected groups treated with artesunate (AS). This highlights AS efficacy against *B. microti* infection in liver and spleen tissues.

### 3.6 Effect of AS treatment and *B. microti* infection on expression levels of key markers of apoptosis and inflammation

To gain deeper insights into the potential effects of artesunate treatment in the context of *Babesia microti* infection, we investigated the mRNA expression levels of several key markers involved in apoptotic processes, and proinflammatory and anti-inflammatory pathways, such as Bax, Beclin 1, Caspase 9, CD44, IFN-γ, IL2, and TNF-α.

**Figure 6:**
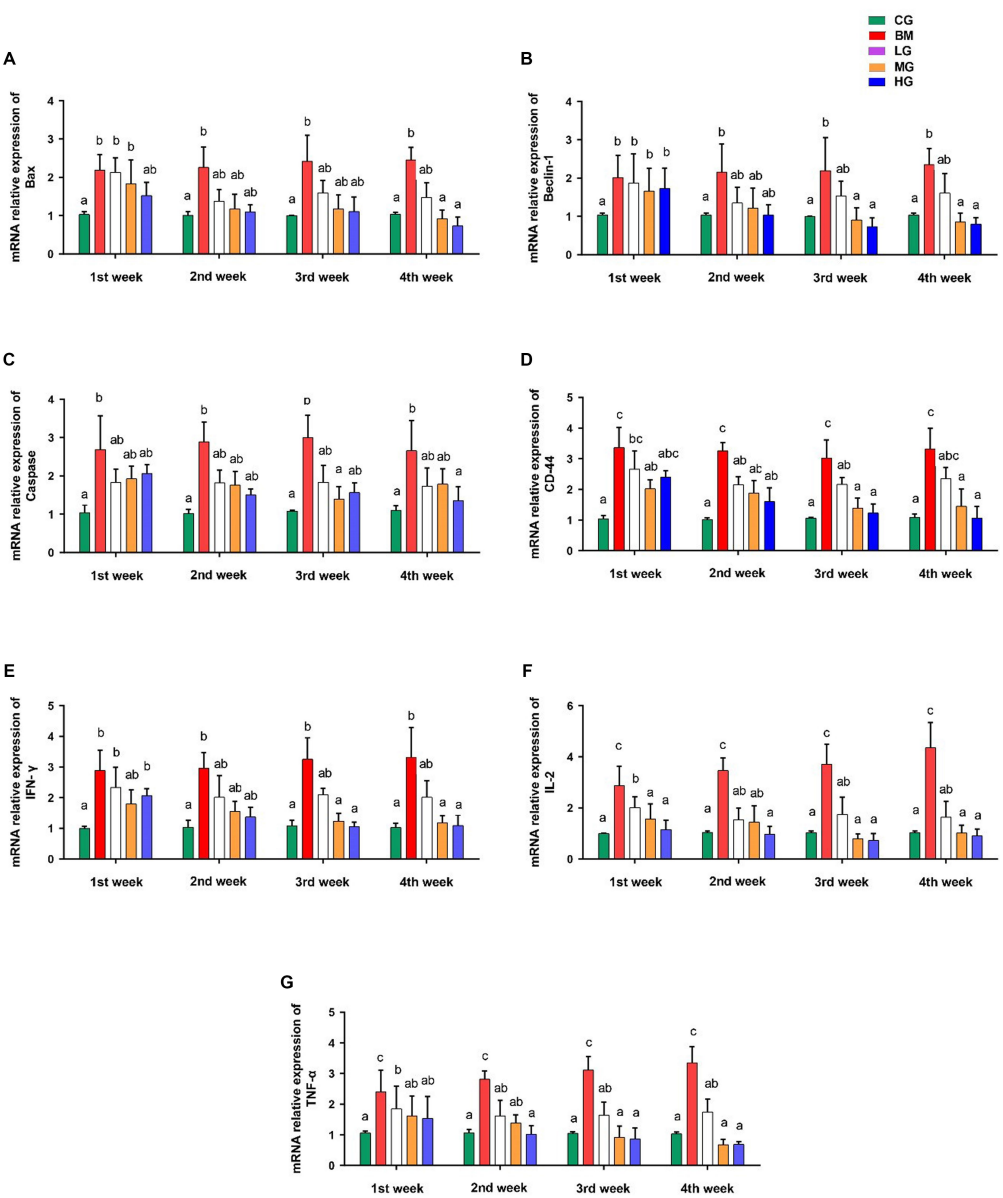
Results of mRNA expression profiles of key markers involved in apoptotic processes and proinflammatory and anti-inflammatory pathways. **(A)** Bax, **(B)** Beclin 1, **(C)** Caspase 9, **(D)** CD44, **(E)** IFN-γ, **(F)** IL2, and **(G)** TNF-α. However, experimental groups including the control group (CG), the *B. microti* group (BM), the *B. microti* + artesunate 2 mg/kg low-dose group (LG), the *B. microti* + artesunate 4 mg/kg medium-dose group (MG), and the *B. microti* + artesunate 8 mg/kg high-dose group (HG). The data were representative of three independent experiments, and the values are presented as the means ± SEM. *p* ≤ 0.05 and *p* ≤ 0.01 were considered significant. The same letters mean non-significant, and the different letters mean significant.

Our findings showed significant changes in mRNA expression levels of key regulatory genes of the apoptotic pathway (Bax, Beclin 1, and Caspase 9) in mice following the administration of AS in a dose-dependent manner. Notably, mice administered with AS at a high dosage of 8 mg/kg (HG) showed a significant reduction in the mRNA expression levels of Bax (Figure 6A), Beclin 1 (Figure 6B), and Caspase 9 (Figure 6C) in comparison with mice that were challenged with *B. microti* (BM) alone (*p* < 0.05). These observations were particularly prominent during the 3rd and 4th weeks post-treatment. However, when AS was administered at lower doses of 2 mg/kg (LG), the expression patterns of these markers closely resembled those of the control group (CG). These findings further emphasize the potential of AS treatment to impact key apoptotic factors, indicating its role in reducing apoptosis induction.

We also investigated what role AS played in mice that were exposed to *B. microti* (BM) to see how it affected the levels of CD44, IFN-γ, IL2, and TNF-α cytokines. When compared to mice that were in (the BM) group and those that were given AS dosages had lower levels of these cytokines. Mice treated with AS at the medium dose of 4 mg/kg (MG) and high dose of 8 mg/kg (HG) exhibited a significant reduction in the expression of CD44 (Figure 6D), IFN-γ (Figure 6E), IL2 (Figure 6F), and TNF-α (Figure 6G) compared to those challenged with *B. microti* alone (BM) (*p* < 0.05). This marked reduction in cytokine expression was evident after the third and fourth weeks of treatment. Conversely, the treatment of mice with AS at a dose of 2 mg/kg resulted in significant changes, although it was not as effective as the treatment at doses 4 and 8 mg/kg (MG and HG) in the expression of these cytokines when compared to the control group (CG).

### 3.7 Effect of AS treatment and *B. microti* infection on immunohistochemistry of apoptosis and inflammation key marker proteins

**Figure 7:**
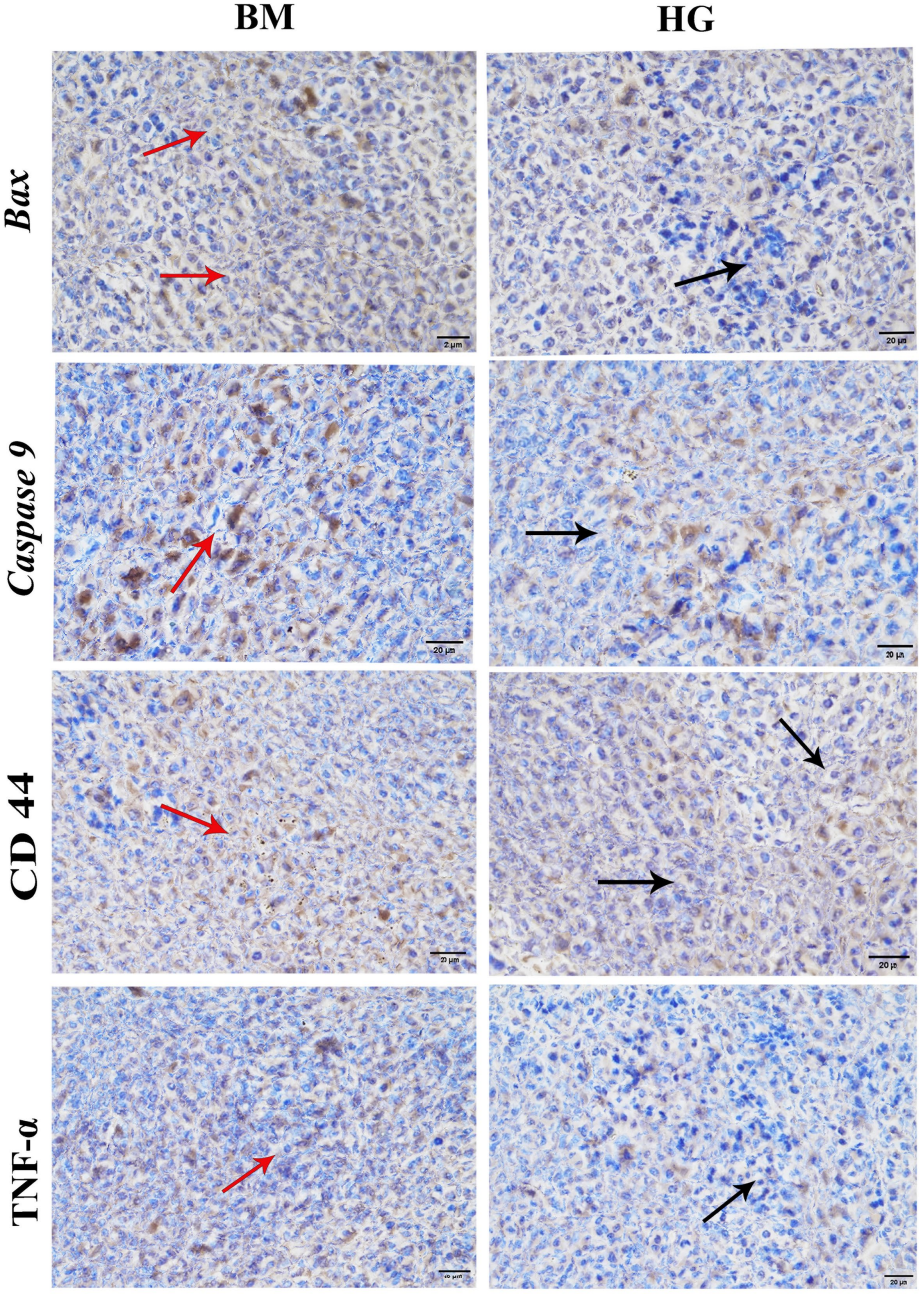
The result of immunohistochemistry analysis comparison between *the B. microti* (BM)-infected group and the *B. microti* + artesunate 8 mg/kg (HG) group. Red arrows indicated positive expression, whereas black arrows showed the absence of detectable proteins. Photomicrograph taken at 400X and scale bar 20 μm.

The levels of important proteins like Bax, Caspase 9, CD44, and TNF-α in both the *B. microti* (BM) and AS treatment groups were detected with immunohistochemistry. In Figure 7, the hepatic cytoplasm of the mice infected with *B. microti* showcased a pronounced dark brown staining, suggesting the presence of considerable positive expression within the cytoplasmic compartment. Conversely, the hepatic cytoplasm exhibited a distinct blue coloration in the mice treated with AS at a dose of 8 mg/kg (HG). This blue hue indicates the absence of detectable protein expression. These results imply that AS effectively counteracts the infection induced by *B. microti* in mice.

### 3.8 Effect of AS on protein expression of apoptosis and inflammation markers

**Figure 8:**
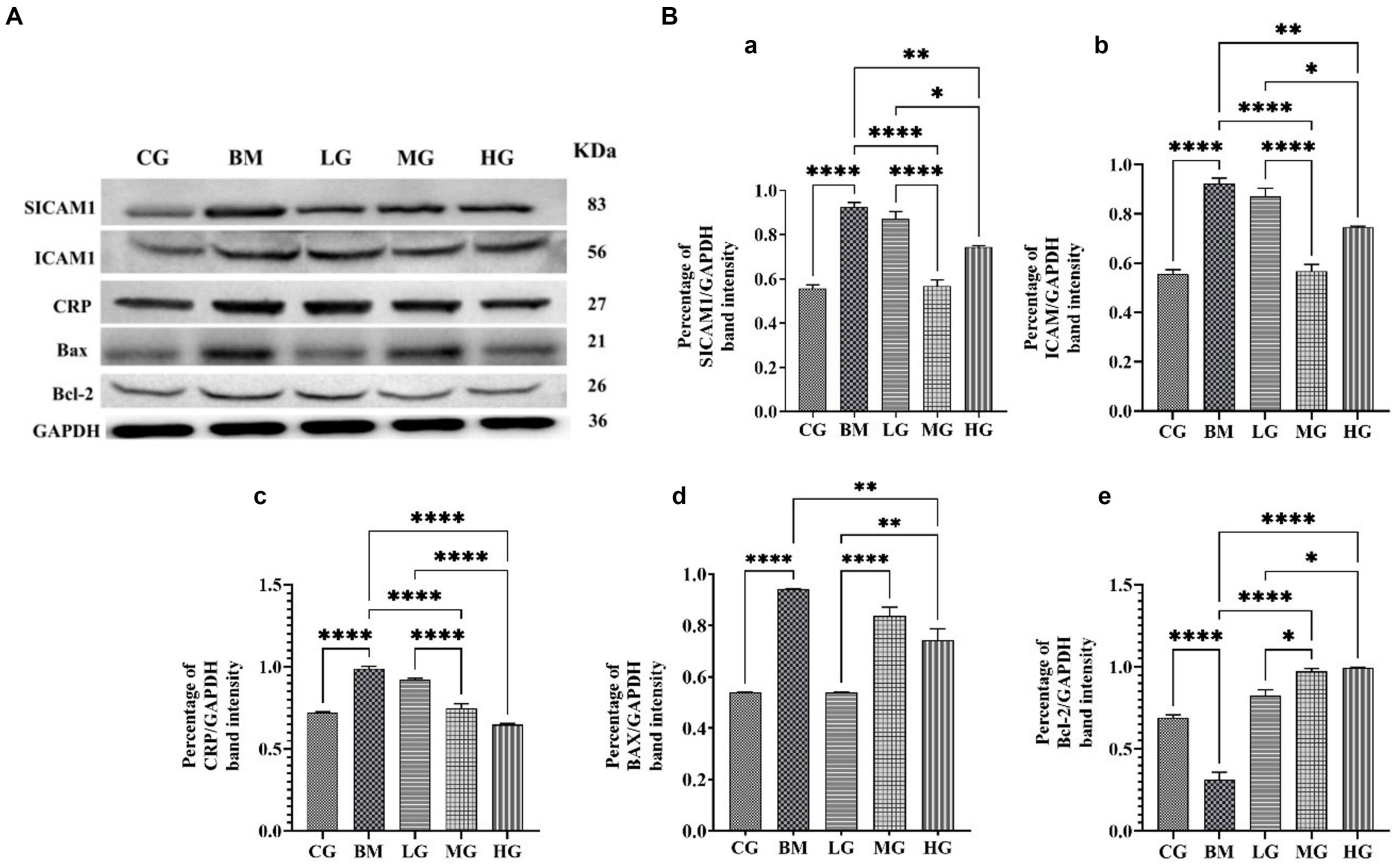
Regulating effect of artesunate (AS) after 4 weeks of treatment. **(A)** Shows protein levels of inflammation markers including SICAM-1, ICAM-1, and C-reactive protein and proapoptotic factor Bax and anti-apoptotic factor Bcl-2. **(B)** Shows a bar graph of the percentage of band intensity relative protein expression compared within the control group (CG), the *B. microti* (BM) group, and the experimental groups *B. microti* + artesunate 2 mg/kg low-dose group (LG), *B*. *microti* + artesunate 4 mg/kg medium-dose group, and 8 mg/kg high-dose group. **(a)** SICAM-1, **(b)** ICAM-1, **(c)** CRP, **(d)** BAX, **(e)** Bcl-2 bar graph, with asterisks representing a higher level of significance, “*” (*p* < 0.05) “**”(*p* < 0.01) ***”(*p* < 0.001)”****”(*p* < 0.0001).

We investigated the expression profiles of two key factors, Bax and Bcl-2, that play important roles in apoptotic pathways, to learn more about how artesunate might help stop apoptosis in *B. microti* infections. Moreover, we also explored the impact of AS on inflammation markers, including SICAM-1, ICAM-1, and C-reactive protein. As shown in Figure 8A. Western blot analysis showed the protein expression of the proapoptotic factor Bax significantly increased, and the anti-apoptotic factor Bcl-2 decreased significantly (*p* < 0.05) in *the B. microti-*infected (BM) group. AS treatment normalizes Bax and Bcl-2 protein expression levels. Intriguingly, the expression pattern in the bar graph, Figures 8B,d,e show a significant (*p* < 0.05) decrease found at 2 (LG) and 8 mg/kg (HG) treatment groups in Bax. However, dose-dependent upregulation of Bcl-2 protein expression was found in AS treatment groups (LG, MG, and HG). This observation highlights the potential of AS treatment to modulate apoptotic processes in *B. microti* infection.

Furthermore, our investigation extended to the realm of inflammation markers. We examined protein expression ICAM-1, SICAM-1, and C-reactive protein levels, and results showed significant (*p* < 0.05) upregulation was found in the BM group as compared to the control (CG) and treatment groups. AS treatment at a low dose of 2 mg/kg (LG), medium dose of 4 mg/kg (MG), and 8 mg/kg (HG) significantly (*p* < 0.05) downregulated the alleviated level in a dose-dependent manner. However, a significant decrease was found at 4 mg/kg in SICAM-1 and ICAM-1 and 8 mg/kg in CRP protein expression.

## 4 Discussion

Animal health is suffering tremendously all over the world due to the spread of parasite infections, and babesiosis is one of the most prevalent diseases that is adversely affecting humans as well as animal populations on a very large scale (26–28). Currently, no drug of choice is available for the cure and treatment of human babesiosis. In animals, only diminazene aceturate is the first line of therapy in cases of severe babesiosis. Nowadays, severe drug resistance has developed against this drug (29). So far, many drugs are under research to find a new novel drug against the Babesia parasite. Artesunate is well known for its antimalarial properties; besides these, it has been extensively studied for antimicrobial, anti-inflammatory, antitumor, immunomodulatory, and antiparasitic properties. This drug is regarded as a potent antimalarial drug throughout the world (9, 30). The causative agents of malaria and babesia shared the same pathobiology and were also misdiagnosed in babesia as malaria (31, 32). So, this study was carried out in different aspects to know the potential effects of artesunate on physiological, serum biochemical, pathological, and immunomodulator response as well as the ameliorative and protective effects of AS against experimentally induced *B. microti* infection in mice.

We found that after a successful infection, it has been noted that *B. microti* has a high impact on body weight, liver, and spleen viscera index, causing weight loss, liver enlargement (hepatomegaly), and spleen enlargement (splenomegaly). This is due to decreased appetite, increased metabolism, red blood cell destruction, inflammation, immune cell infiltration, and heightened macrophage activity (33). A similar finding was observed in the *B. microti*-infected group and those treated with a lower dosage of AS of 2 mg/kg. The inability of the solely *B. microti*-infected group to recover by the 28-day mark underscores the severity of the infection. In contrast, the ameliorative effects observed in the AS-treated groups point toward its potential to mitigate the impact of the infection on growth and development. This trend becomes particularly evident with increasing AS dosages (4 mg/kg and 8 mg/kg), whereas dose-dependent improvement in body weight and organ indices is evident. The same finding was observed in experimentally induced leishmania parasites in mice at 37 days post-treatment significant increase in body weight and recovered state of liver and spleen viscera index with combined therapy of diminazene and chloroquine therapy (34).

*B. microti* is an intra-erythrocytic parasite that mainly infects red blood cells causing fever and severe forms of anemia, it gets more attention from researchers due to its zoonotic importance. It can readily transmitted from infected rodents and small pet animals to humans (2, 35). We found treatment with AS at a higher dosage of 8 mg/kg yielded notable findings regarding the modulation of hematological parameters. The hematologic implications of AS administration are pronounced. The dose-dependent elevation in red blood cell (RBC), white blood cell (WBC), and platelet (PLT) counts consequent to AS treatment illuminate its role in positively impacting hematopoietic processes. This finding holds substantial clinical relevance, suggesting that AS treatment could potentially contribute to restoring hematologic homeostasis disrupted by *B. microti* infection. These findings are consistent with previous research in which patients infected with *Plasmodium falciparum*, following treatment with AS, exhibited a rapid recovery of vital hematological parameters. On the 42nd day post-treatment, a substantial restoration was observed in red blood cell (RBC) counts, white blood cell (WBC) counts, hemoglobin levels, and platelet counts. This resurgence in hematological indices underscores the efficacy of AS in promoting recovery and re-establishing normalcy in individuals afflicted by *Plasmodium falciparum* infection (36).

*B.microti* infection leads to abnormal liver function due to hepatocellular injury hemolysis and bile duct blockage leading to increased production of liver enzyme alanine transferase (ALT), alkaline phosphatase (ALP), bilirubin, and total protein count (37). In this context, we investigated AS treatment at varying dosages (2 mg/kg, 4 mg/kg, and 8 mg/kg) on mice with a focus on key biochemical parameters. Notably, the findings revealed significant reductions in the levels of alanine transaminase (ALT), alkaline phosphatase (ALP), and total protein in mice subjected to AS treatment when compared to the *B. microti*-infected group. Mice within the infection group displayed a significant increase in the concentration of aspartate transaminase (AST) throughout the study. Additionally, minor increases were observed in the levels of ALT, ALP, bilirubin, and total protein in this group when compared to the control group at various time points (1st, 2nd, 3rd, and 4th weeks). These observations underscore the biochemical alterations induced by *B. microti* infection, emphasizing the impact of the disease on liver function and protein metabolism. Furthermore, the animals treated with artesunate dosages of 2 mg/kg, 4 mg/kg, and 8 mg/kg exhibited a minor decrease in the levels of ALT, ALP, AST, bilirubin, and total protein when compared to the *B. microti*-infected group. This suggests that artesunate treatment may have a potential ameliorative effect on these biochemical parameters, attenuating the adverse consequences of *Babesia microti* infection. This study was in agreement with Abiodun (38).

The *B. microti* parasite inoculates the liver through the central hepatic vein and the spleen through the splenic artery. After entry, serous congestion of red pulp and proliferation of megakaryocytes lead to an acute phase of infection (33). In a previously conducted comparative study, the effect of artesunate and chloroquine treatment on histopathological damages caused by *Plasmodium berghei* in albino mice showed severe damage to the liver and spleen that was significantly reduced by artesunate treatment as compared to chloroquine (39). Similarly, we observed a dose-dependent manner recovery of spleen cellular structure with weak red pulp cellularity and white pulp with clear marginal zones around follicles. Hepatic lobules and central vein are unformed in appearance in the control and at high dosage (8 mg/kg) groups to show AS possesses potent effects of antiparasitic against *B. microti* infection.

Beyond its direct antiparasitic properties, AS may have additional therapeutic benefits due to its capacity to regulate the expression of apoptotic proteins Bax, Beclin, and Caspase activity through the destruction of contaminated cells, stop the infection from spreading, and encourage tissue regeneration (40). Recently, documented studies on 56 phytochemicals including AS showed significant reduction in TNF-α, IL2, CD44, and IFN-γ via NF-kB pathways *in vitro* and *in vivo* models (41). In our study, mice administered with AS at a dosage of 8 mg/kg showed a significant reduction in the mRNA expression levels of Bax, Beclin 1, and Caspase 9 in comparison to mice that were challenged with *B. microti* alone (Figure 6). Mice treated with AS at the medium dose of 4 mg/kg and high dose of 8 mg/kg exhibited a significant reduction in the expression of CD44, IFN-γ, IL2, and TNF-α compared to those challenged with *B. microti* alone. This modulation suggests a complex interplay between AS and cellular responses, potentially influencing the balance between cell survival and apoptosis.

The distinctive staining patterns observed in immunohistochemistry analysis showed hepatic cytoplasm between the *B. microti-*infected and AS at a higher dosage of 8 mg/kg showed the hepatic cytoplasm of the mice infected with *B. microti* showcased a pronounced strong tissue immune expression of Bax, Caspase 9, CD44 and TNF-α staining, underscoring the presence of considerable positive expression within the cytoplasmic compartment. Conversely, the hepatic cytoplasm exhibited a distinct blue coloration in the mice treated with AS at a dose of 8 mg/kg. This blue hue indicates the absence of these detectable proteins Bax, Caspase 9, CD44, and TNF-α expression, this underscores the potential of AS treatment to influence protein expression dynamics within a cellular context. These findings are consistent with previous research that AS suppresses the oxidative and inflammatory processes by activating nuclear factor erythroid 2-related factor that inhibits caspase activity and reduces the apoptosis regulator Bax and Bcl-2 expression ratio by activating Nrf2 and downregulating ROS-dependent p38 mitogen-activated protein kinase in mice (42).

Babesiosis disrupts the delicate balance between apoptosis and inflammation, ICAM-1, a cell adhesion molecule, facilitates the interaction between leukocytes, immune cells, and endothelial cells, which line the blood vessels (43). CRP, a non-specific inflammatory marker produced by the liver, is used to assess the presence and severity of inflammation. SICAM-1, soluble form of ICAM-1, shed from the cell surface into the bloodstream provides a more sensitive and non-invasive measure of inflammation compared to ICAM-1 inflammatory mediators that are upregulated in response to this disturbance, as well as pro- and anti-apoptotic proteins such as Bax, a proapoptotic protein, which plays a critical role in initiating apoptosis by inducing mitochondrial damage and releasing cytochrome c (44). Our Western blot analysis showed a marked significant increase in expression levels of key apoptotic proteins Bax, and Bcl-2 expression decreased significantly (*p* < 0.05) in *the B. microti-*infected group, AS normalized Bax and Bcl-2 expression ratio. However, expression of inflammatory proteins (ICAM-1, SICAM-1, and C-reactive protein) was upregulated in the *B. microti*-infected group and AS treatment significantly (*p* < 0.05) downregulated this alleviation in a dose-dependent manner. This is a correlation with the results of De Mast et al. (43) and Park et al. (45) who noted elevated vWf, VCAM-1, SICAM-1, and C-reactive protein levels in children who have a microscopic asymptomatic malarial illness. This suggests that endothelial cells could be among the early host responses to the presence of parasites in the case of parasitemia. Our study results are also in agreement with the findings of Baric et al. (46) who checked ICAM-1, SICAM-1, and CRP levels through ELISA kit in dog blood samples suffering from canine babesiosis. These parameters were noted elevated before treatment but returned to normal after treatment which indicates the promising effects of AS for babesiosis treatment. The AS administration underscores the intricate role in regulating apoptotic pathways and alleviating inflammatory response during *B. microti* infection.

## 5 Conclusion

In conclusion, the present study provides potential evidence that AS may have a promising effect on *B. microti* infection by increasing body weight, recovering liver and spleen index state in mice, improving red blood cell (RBC), white blood cell (WBC), and platelet (PLT) counts, and restoring blood homeostasis, downregulating levels of alanine aminotransferase (ALT), aminotransferase glutamate (AST), alkaline phosphatase (ALP), and total protein; restoring cytoarchitectural damage in the liver and spleen; modulating the pathway of cellular apoptosis and attenuating in inflammatory response, and in a dose-dependent manner. Furthermore, these insights contribute significantly to understanding AS therapeutic mechanisms and their potential utility as an adjunctive treatment for Babesia infections.

## Data availability statement

The raw data supporting the conclusions of this article will be made available by the authors, without undue reservation.

## Ethics statement

The animal study was approved by the Institutional Research Ethics Committee, Northeast Agricultural University. The study was conducted in accordance with the local legislation and institutional requirements.

## Author contributions

SF: Writing – original draft, Software, Methodology, Investigation, Formal analysis. WA: Writing – review & editing, Resources, Investigation, Formal analysis. SiL: Writing – review & editing, Validation, Project administration, Resources, Investigation. MH: Writing – review & editing, Validation. MI: Software, Data curation, Writing – review & editing. ShL: Validation, Formal analysis, Writing – review & editing, Software. MF: Methodology, Writing – review & editing, Software, Formal analysis. MS: Data curation, Writing – review & editing, Software, Formal analysis. XZ: Validation, Supervision, Project administration, Funding acquisition, Conceptualization, Writing – review & editing.
